# The Role of EU State Aid Law as a “Risk Management Tool” in the COVID-19 Crisis

**DOI:** 10.1017/err.2020.71

**Published:** 2020-07-27

**Authors:** Delia FERRI

**Affiliations:** * Professor of Law, Maynooth University Department of Law, Ireland; email: delia.ferri@mu.ie.

## Abstract

This article discusses the role of European Union (EU) State aid law in the COVID-19 crisis. It contends that different Treaty derogations have played unique roles in addressing the core determinants of the economic risk linked to the pandemic (ie the “exposure” to lockdown measures and the “vulnerability” of certain sectors to them), and in increasing the resilience of national economies. Moreover, this article examines the extent to which EU State aid law has been used to manage and mitigate health risks, by allowing Member States to enhance the preparedness and capacity of their healthcare sector (broadly conceived) to respond to the pandemic. On the whole, this article maintains that State aid control has been used by the European Commission as an important “risk management tool”, and it highlights the role of the Commission as the crisis management authority.

## I. Introduction

COVID-19 has had a staggering impact on our lives. It has changed how we work, learn and enjoy our free time. At the time of writing, schools and universities throughout the European Union (EU) are closed, forcing teaching and assessment activities to continue online. Several sports tournaments have been cancelled or postponed, and most workplaces are closed with employees working (if possible) from home. This unprecedented health crisis, which “requires management of a catastrophe which shatters societies”,^1^ has also given rise to a range of socioeconomic side effects that, at present, are difficult to quantify, but will most likely cause the deepest recession in European history.^2^ Among the plethora of measures that the EU has resorted to in order to effectively respond to the COVID-19 pandemic lies State aid control.^3^ Garicano suggests that the European Commission “has decided that Europe will not stand in the way of EU Member States’ responses”.^4^ However, it seems that the Commission has done far more than that. Since the beginning of March, it has promptly used its powers in the field of State aid to allow Member States to inject substantial amounts of public money into their economies. It has reacted faster in this field than in others, facilitating the quick deployment of national funding (while slowly trying to mobilise EU resources)^5^ and approving national measures, as mentioned by Biondi, “at the speed of light”.^6^ Notably, Member States even waived their linguistic rights in order to facilitate the swift adoption of decisions (in English) by the Commission.^7^

It is well known that State aid is, in principle, prohibited by Article 107(1) TFEU, which provides that any aid granted by a Member State or through State resources that distorts, or threatens to distort, competition by favouring certain undertakings or the production of certain goods is incompatible with the internal market, insofar as it affects trade between the Member States.^8^ However, in spite of this blanket prohibition, as recalled by the Commission in its Communication released in mid-March,^9^ the EU State aid regime allows Member States to intervene in their economy. Member States can enact measures of general application (which are not selective), such as generalised de-taxation measures, and/or measures in the form of general wage subsidies, or measures directly aimed at supporting consumers, as they fall already outside the scope of Article 107 TFEU. Furthermore, the blanket prohibition included in Article 107(1) TFEU is allayed by a set of derogations laid down in Article 107(2) and (3) TFEU. These paragraphs recognise that an economic intervention from the State may be needed in specific circumstances, or to address well-defined market failures and to protect certain social values. Article 107(2) and (3) TFEU entail that Member States can adopt aid measures within the remit of these Treaty exceptions further to the Commission’s approval.^10^ In addition, the General Block Exemption Regulation (GBER) permits Member States to intervene in their economy with no need to prior notification to the Commission.^11^

This regime is, however, inadequate in exceptional times, as it does not allow massive amounts of State aid. Hence, at the end of March 2020, and based on the experience acquired with a similar instrument during the 2008 financial crisis, a “Temporary Framework for State aid measures to support the economy in the current COVID-19 outbreak” (Temporary Framework) was approved by the Commission.^12^ This Temporary Framework is aimed to ensure a “targeted and proportionate application of EU State aid control”, which guarantees that “national support measures are effective in helping the affected undertakings during the COVID-19 outbreak”.^13^ It recognises that “the COVID-19 outbreak affects the whole EU and that the containment measures taken by Member States impact undertakings” causing a serious disturbance to the economy.^14^ As Buendia Sierra and Dovalo point out,^15^ the Temporary Framework is not a hard law instrument: consequently, it does not change existing rules, nor does it set directly applicable rules. Yet, in outlining the conditions under which a Member State is authorised to enact State aid measures, it substantially enhances the breadth of the derogations provided for in the Treaty. Moreover, it gives legal certainty as to the approach that the Commission adopts during this crisis. The Temporary Framework will be applied to all notified measures until 31 December 2020, but the Commission reserves for itself the right to periodically revise it “on the basis of important competition policy or economic considerations”.^16^ In fact, as it will be further discussed in the remainder of this article, at the time of writing, the Temporary Framework has already been modified (ie extended) twice: firstly, at the beginning of April,^17^ and then on 8 May 2020.^18^ On 12 June 2020, the Commission launched a new consultation with Member States to further extend the scope of the Temporary Framework, in order to support micro and small enterprises – including start-ups – that were already in difficulty before the outbreak and that have been heavily hit by the crisis.^19^ The Commission also proposes to provide incentives for private investors to participate in recapitalisation measures “to encourage capital injections with significant private participation also in private companies, limiting the need for State aid and the risk of competition distortions”.^20^

Against this background, this article discusses the role of EU State aid law in the COVID-19 crisis. Instead of commenting on the Temporary Framework as such or describing the State measures adopted so far, this article adopts a systematic approach and a specific conceptual standpoint – that of “risk”. In particular, it discusses the extent to which the Commission has sought, through State aid control, to reduce the “economic risk” linked to lockdown measures adopted by Member States and to mitigate health risks. With regard to the former aspect, this article looks at the core determinants of the “economic risk” arising from the COVID-19 outbreak. As suggested by Noy et al, those determinants are not only the virus itself (ie the “hazard”), but also the “exposure to it” (or, better, to the lockdown measures adopted to contain it), and the “vulnerability” of certain sectors to it.^21^ Noy et al also submit that the economic risk is diminished where the resilience of the economy is increased.^22^ In a similar vein, this article maintains that different Treaty derogations play unique roles in mitigating the “economic risk” stemming from the pandemic by acting on those determinants. “Exposure” is conceived of as the direct losses and damages experienced by undertakings further to lockdown measures. Vulnerability, in this analysis, is determined by a reduced liquidity arising from the decrease in demand due to the pandemic in combination with factors inherent in the way in which that sector is organised. Furthermore, as in the analysis proposed by Noy et al, resilience is conceptualised as “the ability of the economy to bounce back”.^23^ With regard to the second aspect, this article focuses on the role of EU State aid law in relation to health risks (infection rates, spread of the virus, mortality rates). In that connection, it examines the extent to which the Commission has used State aid control to enhance the preparedness and capacity of the national health sectors to respond to the pandemic, treat the infectious disease and limit the contagion.

On the whole, this article contends that EU State aid law has played a pivotal role in a situation of economic downturn, exactly as it happened during the financial crisis, and has proven important to addressing the risks brought about by the pandemic. However, this article does not engage in a comparison between the approach adopted by the Commission in 2008 and the current one. Rather, it aims to use the concept of “risk” as lens through which to analyse State aid practice in the COVID-19 period. While acknowledging that State aid control is not a panacea and cannot substitute regulatory instruments (especially when it comes to health risks), this article suggests that the State aid toolbox has successfully been deployed by the Commission as an important “risk management tool”. It ultimately highlights the role of the Commission as the “*de facto* crisis-management authority”.^24^

## II. The role of EU State aid law in reducing the economic risk

The sudden economic decline caused by the COVID-19 outbreak, colloquially referred to as the “black swan of 2020”,^25^ has created a “state of emergency” in the EU. It has not only given rise to unprecedented restrictive measures on free movement,^26^ but has also prompted an unparalleled level of subsidisation. Thus far, through the application of EU State aid rules, the Commission has allowed Member States to tackle the economic risk linked to the pandemic. Article 107(2)(b) TFEU has proven to be an essential tool to reduce “exposure”, while Article 107(3)(b) TFEU, in light of the Temporary Framework, has allowed for a wide array of national measures aimed at addressing the “vulnerability” of certain economic sectors and at stimulating their resilience.^27^

### 1. The role of EU State aid law in remedying “exposure”

At the outset of the crisis, Article 107(2)(b) TFEU was immediately identified as the primary derogation to facilitate the swift adoption of national aid measures. It is well known that Article 107(2) TFEU specifies a number of instances in which State aid is permissible (*de jure* derogations). The Commission does not enjoy any discretionary power in respect of aid assessed under this provision. If it verifies that the conditions laid down in the Treaty are met, the Commission considers the aid falling under the *de jure* derogations compatible with the internal market. Nonetheless, in ascertaining that those conditions are met, it exercises its interpretive authority. In particular, Article 107(2)(b) TFEU allows for “aid to make good the damage caused by natural disasters or *exceptional occurrences*”.^28^ This provision entails that aid is allowed if three conditions are met: firstly, the event, further to which the aid is granted, qualifies as a natural disaster or exceptional occurrence; secondly, there is a direct link between the damage and the natural disaster or exceptional occurrence; and thirdly, the aid cannot overcompensate for the damage that occurred. In relation to the first (and preliminary) condition, while natural disasters encompass earthquakes, avalanches, landslides, floods and other adverse climatic conditions,^29^ the categorisation of an event as an exceptional occurrence has always been made by the Commission on a case-by-case basis.^30^ The Commission qualified as exceptional occurrences war, internal disturbances or strikes or major nuclear or industrial accidents,^31^ and all of those events that fulfil three cumulative criteria: firstly, they are unforeseeable (or extremely difficult to foresee); secondly, they have significant economic impact; and thirdly, they are “extraordinary”. Notably, in the past, the Commission considered the outbreak of the bovine spongiform encephalopathy (BSE) disease as an exceptional occurrence.^32^ With regard to the second condition, the damage can be provoked by a “physical loss” (ie to physical destruction of facilities or death of livestock) or can entail a financial loss, as long as this is attributed solely to the natural disaster or to an exceptional occurrence.^33^ In order to avoid overcompensation, “as precise an assessment as possible must be made of the damage suffered by the producers concerned”.^34^

Having regard to its previous practice, the Commission could determine that the COVID-19 outbreak was not foreseeable. It argued that the World Health Organization (WHO) declaration of a pandemic, “associated with the public health risk deriving from the absence of therapeutics or vaccines for the novel COVID-19 determine the exceptional nature of the circumstances”.^35^ It also acknowledged that the swiftness of the spread of this virus, as well as the necessity to “adopt and encourage the respect of measures aimed at interrupting transmission chains”, will result in a far-reaching disruption of various economic sectors, which is “clearly outside the normal functioning of the market” (ie extraordinary).^36^ The Commission qualified the current COVID-19 epidemic as an exceptional occurrence for the purpose of Article 107(2)(b) TFEU in early March, in a decision adopted overnight.^37^ Then, it generally found that all aid measures notified were compensating (part of) damages directly linked to the COVID-19 pandemic, which were suffered by relevant economic operators. The Commission has also considered that all measures were proportionate.

It seems evident that Article 107(2)(b) TFEU has proven to be the primary avenue to address the economic risk linked to the “exposure” of certain sectors of the economy to the immediate and detrimental effects of COVID-19 lockdown actions adopted by national governments by redressing the damages incurred by economic operators. The earliest and primary example of aid approved under Article 107(2)(b) TFEU is the Danish scheme to compensate damage caused as a result of the cancellation of large public events due to the COVID-19 outbreak,^38^ which was notified by the Danish government on 11 March 2020 and rapidly approved by the Commission. Under that scheme, worth €12 million, economic operators would be entitled to compensation for the losses suffered as a consequence of the cancellation or postponement of public events, for which tickets were already sold. A similar scheme was notified to the Commission by the Swedish government and was approved in April 2020.^39^ The latter scheme (worth €38 million) was targeted at cultural events that were cancelled or pushed back. Other more general measures addressed the significant damages caused to companies by “exposure” to the COVID-19-related lockdown, such as the Danish scheme to support all of those businesses that “have been impacted by … containment measures some of which, more immediately and *directly exposed*, faced a massive decline in demand leading to a partial or full non-utilization of their facilities or services”.^40^ Other examples are the €650 million Dutch scheme intended to compensate companies in the floriculture, horticulture and potato sectors for the loss of revenue or additional costs related to the collapse in demand for their products,^41^ as well as the Austrian scheme to compensate for the economic damage resulting from the containment and lockdown measures and suffered by companies.^42^ A handful of measures to compensate the losses of revenues to certain airlines, caused by “exposure” to temporary travel bans or the limitation of free movement, were also speedily approved by the Commission.^43^ This is the case for the €550 million public loan in favour of the German airline Condor,^44^ of the guarantee for Scandinavian Airlines,^45^ of the French scheme deferring payment by airlines of certain taxes,^46^ and of the most recent aid to Austrian Airlines.^47^

Exposure to COVID-19 lockdown measures appears sufficiently counterbalanced by schemes intended to compensate businesses’ damages (including both *damnum emergens* and *lucrum cessans*) approved under Article 107(2)(b) TFEU. As noted in the Temporary Framework, under this derogation, Member States could also provide compensation to those companies that had already received “rescue aid, restructuring aid or temporary restructuring support” under Article 107(3)(c) TFEU within the meaning of the “Rescue and Restructuring Guidelines”.^48^ Therefore, the Commission, in assessing aid under Article 107(2)(b) TFEU, has given Member States wide leeway to tackle the current losses suffered by companies and to provide them with the liquidity necessary to afford certain fixed costs. However, as contended by Nicolaides, it might be difficult “to disentangle the impact of the exceptional occurrence from other factors in the economy”,^49^ and more so when the pandemic will be over. The derogation under Article 107(2)(b) TFEU is, hence, of use for losses caused primarily and directly by the exposure to COVID-19 during the peak of the pandemic or in its immediate aftermath. Moreover, compensation measures alone are not capable of addressing the full impact of COVID-19 on the economy of a Member State. They need to be combined with public support schemes that mitigate specific vulnerabilities and enhance the resilience of many economic sectors by injecting liquidity that goes beyond the compensation of direct damages while increasing trust in the overall macroeconomic stability.

### 2. The role of EU State aid law in reducing “vulnerability” and increasing “resilience” of different economic sectors

Given that compensation measures are *ictu oculi* insufficient to address the economic risks linked to the pandemic, it almost immediately became evident that Article 107(3) TFEU was to be invoked.

Under Article 107(3)(b) TFEU, the Commission can declare compatible with the internal market aid aimed “to remedy a serious disturbance in the economy of a Member State”. Existing rules concerning the application of this derogation would have permitted the adoption of an array of measures.^50^ However, the Commission acknowledged the need to boost the potential for Article 107(3)(b) TFEU to allow for heavy State intervention within national economies, at the beginning referring only to the situation in Italy and Spain,^51^ and subsequently to that of all EU Member States. In order to ensure a coherent application of Article 107(3)(b) TFEU, the Temporary Framework was enacted. This Temporary Framework, by offering a clear (and looser) interpretation of Treaty rules, gives Member States a wider lineal space within which to address liquidity shortages. It also provides a uniform and pan-European roadmap for national funding, allowing for better management of the economic risk linked to COVID-19.

The Temporary Framework is premised on the assumption that “[w]ell-targeted public support is needed to ensure that sufficient liquidity remains available in the markets, to counter the damage inflicted on healthy undertakings *and to preserve the continuity of economic activity during and after the COVID-19 outbreak*”.^52^ In that connection, it allows for aid in the form of direct grants, repayable advances or tax advantages under the threshold of €800,000 per undertaking, or €120,000 per undertaking active in the fishery and aquaculture sector, and €100,000 per undertaking active in the primary production of agricultural products. In order to ensure that companies have access to adequate liquidity, it permits public guarantees on loans for a limited period,^53^ as well as subsidies to public loans,^54^ and it sets out a series of criteria that those guarantees must fulfil. Aid in the form of public guarantees and reduced interest rates can also be provided through credit institutions and other financial intermediaries.^55^ The Temporary Framework introduces additional flexibility on short-term export credit insurance and enables Member States to support such insurance where needed.^56^ It also establishes that the Commission will consider as compatible with the internal market on the basis of Article 107(3)(b) TFEU aid measures that provide for temporary deferrals of taxes or of social security contributions with regard to undertakings particularly affected by the COVID-19 outbreak.^57^ Finally, the Temporary Framework addresses the social impact of this sudden economic downturn, and in order to prevent a massive rise in unemployment, it allows Member States to enact specific aid measures in certain sectors or with regard to specific undertakings to cover the wage costs of these undertakings (including self-employed individuals).^58^ Those wage subsidies must be granted over a period of no more than twelve months and shall not exceed 80% of the monthly gross salary of the benefitting personnel.^59^

The identification of aid ceilings and clear criteria aims to ensure that national aid measures are proportionate and do not unnecessarily distort competition. It is evident that the Commission has estimated the proportionality of State aid *ex ante*, on the basis of its long-standing practice and the experience gained in 2008. Nonetheless, it is also apparent that, during this crisis, State aid rules have prioritised reducing the vulnerability and supporting the resilience of the economy over avoiding distortions to competition. At present, amidst a crisis whose contours are rather uncertain, it is difficult to evaluate whether the current framework is truly “proportionate” and whether stricter rules (and lower ceilings) would have tamed the economic risk brought by COVID-19 in a similar way, with arguably fewer distortions. However, the Commission’s intention to revise the Framework periodically and, seemingly, to come back to a stricter level of scrutiny after this economic storm should ensure respect for the principle of proportionality. In that connection, it seems likely that State support would be gradually withdrawn in 2021, as soon as market conditions improve.

As yet, a large number of schemes, approved by the Commission under the Temporary Framework, address the “vulnerability” of various economic sectors determined by a lack of liquidity, mostly in the form of guarantees and loans.^60^ Interestingly, a few schemes aim to support small and medium enterprises (SMEs),^61^ recognising that they are particularly vulnerable during the COVID-19 outbreak and, in a disrupted demand–supply chain, are most likely to be acutely affected. It appears that aid to SMEs exempted from notification under the GBER is no longer a viable option given the large amount of public money to be injected. Among those measures, the Italian State guarantee to support a debt moratorium by banks to SME borrowers^62^ and the Danish guarantee scheme for SMEs^63^ were approved at the beginning of the crisis. Several other schemes were approved between April and July 2020,^64^ such as two Belgian-Flemish loan schemes for SMEs and start-ups,^65^ a French guarantee for SMEs with export activities that are affected by the coronavirus outbreak^66^ and a Bulgarian scheme worth €88 million to support micro and small enterprises.^67^ The latter scheme tallies with the Bulgarian public guarantee scheme to support SMEs in the context of the coronavirus outbreak (the so-called “Intermediated SME Loan Guarantee Program”). Furthermore, the Irish “Restart Grant” scheme addresses “liquidity shortage faced by micro and small enterprises because of the outbreak”, and aims to ensure their viability and economic continuity.^68^ In a similar vein, the Netherlands enacted a new scheme for public guarantees on small individual loans from banks or other financial institutions to SMEs to support enterprises in the current COVID-19 outbreak and facilitate their access to external finance in a disrupted credit market,^69^ alongside a direct grant scheme to support the fixed costs for SMEs.^70^ These were further complemented by general schemes targeting both SMEs and large enterprises.^71^ Moreover, to mitigate the risk liked to the particular vulnerability of SMEs, national schemes are accompanied by pan-European liquidity measures. In that connection, the Commission has mobilised financial support for SMEs through the Programme for the Competitiveness of enterprises and SMEs (COSME) 2014–2020,^72^ which is worth €2.3 billion. While COSME has been deemed successful overall in the mid-term evaluation,^73^ its effectiveness for enhancing access to finance at this critical time remains unclear. The combination of State aid and EU funding for SMEs is fundamental to addressing the well-known difficulty in accessing finance, which has only been exacerbated during the pandemic and that SMEs have faced since the 2008 crisis.^74^ The use of the Temporary Framework is consequently in line with the Union’s broader policies in this field. The Commission is well aware that those measures are ultimately vital to the survival of the whole internal market, given that SMEs are the “backbone” of the EU economy and account “for 99.8% of total enterprises”^75^ and “for 85% of new jobs created in the last five years”.^76^ The achievement of the EU objective of reinforcing the role (and competitiveness) of SMEs is conditional on their businesses remaining viable during the pandemic. This has been made evident by the latest Commission proposal to expand the Temporary Framework to further support micro-enterprises and SMEs, as well as start-up companies.^77^ In that regard, Commissioner Vestager reiterated that SMEs “are crucial for the economic recovery of the Union”.^78^ It therefore seems that State aid is only one of the tools within the overall Commission policy on SMEs, and it fits well with the general objectives of improving market access and access to financing within the SME Strategy for a sustainable and digital Europe.^79^ After the peak of the pandemic, it will also be important for the Commission to nudge State aid towards supporting (more overtly) digitisation and committing to the sustainability of those enterprises.

Remarkably, some of the measures approved specify that the beneficiaries of the schemes must reside for tax purposes in the European Economic Area (EEA). This is the case, for example, of a Polish aid in the form of a repayable advance to undertakings.^80^ This scheme specifies that the main beneficiary of the aid cannot have tax residence in “tax havens” (which includes, among others, Cayman Islands, Panama, Seychelles and US Virgin Islands).^81^ In its assessment, The Commission noted “that the clause according to which beneficiaries of this scheme need to be tax residents in the EEA … does not constitute a breach of intrinsically linked internal market rules because of its non-discriminatory effect”.^82^ Still, Member States cannot exclude from the beneficiaries of the aid schemes companies with a tax residency in a different EU country, as this would undermine the internal market. However, the approach adopted by the Commission is noteworthy in that it allows Member States not only to tackle COVID-19-related “vulnerabilities”, but also to achieve tax policy objectives and discourage tax evasion and tax avoidance. While this remains outside the scope of this article, this decision confirms the indissoluble link between State aid and fiscal policies.^83^

The Temporary Framework, whilst proving to be a vital instrument in addressing the risk liked to vulnerability, has not (yet) allowed Member States to tackle the increased weakness of undertakings that were already (and for various reasons) in a difficult position, long before the COVID-19 outbreak. In that vein, the Commission has sought to limit the potential for Member States to use the Framework as a smokescreen for indiscriminate subsidisation that might further endanger the already precarious status of the internal market. It intended to discourage national measures not necessarily prompted by the pandemic and to ensure that national governments only use aid with the view to addressing economic risks directly connected to the COVID-19 outbreak. Nonetheless, it transpires that the Commission has approved national aid to rescue companies on the verge of collapse and whose viability was questionable long before December 2019, after having assessed it under Article 107(3)(c) TFEU within the scope of the “Rescue and Restructuring Guidelines”.^84^ This is the case for the Portuguese €1.2 billion rescue loan in favour of *Transportes Aéreos Portugueses* (TAP), which should allow TAP to address its current liquidity needs.^85^ The Portuguese authorities have ensured that TAP will repay the loan granted or release a restructuring plan within six months. However, interestingly, the Commission continued to justify the necessity and proportionality of the measure, taking into account its potential to avoid disruptions for passengers in view of the easing of travel restrictions, and it considered that it indirectly supports the Portuguese tourism sector, which has been hit hard by the coronavirus outbreak. It remains to be seen whether this loose approach (which arguably is questionable in terms of protecting competition in the internal market) will be adopted in other cases, such as the tricky Alitalia renationalisation, which largely predates the COVID-19 crisis and is the latest of numerous rescue operations, and on which the Commission has opened an in-depth investigation.^86^ Certainly, the new proposal under discussion, which would enable, if approved, Member States to support at least certain small enterprises that were already in difficulty before the cut-off date of 31 December 2019,^87^ might contribute to supporting an even more relaxed approach to rescue packages for larger vulnerable companies.

A few measures aimed primarily at increasing “resilience” and allowing undertakings to bounce back more easily after the crisis have also been approved under the Temporary Framework. These include, for instance, schemes that secure rent relief for companies. An example is the Lithuanian aid in the form of direct grants to compensate part of the rent for the undertakings most affected by the COVID-19 outbreak, especially for those whose activities were banned during the quarantine period.^88^ Similar measures was adopted in Slovenia,^89^ Estonia, Czech Republic and Slovakia.^90^ These measures tally with other schemes to ensure that undertakings have sufficient liquidity to remain in the market and to preserve the continuity of economic activity after the COVID-19 outbreak. While rent relief schemes for commercial tenants might be a good option in the short term and might effectively increase the resilience of strained commercial sectors, their viability in the medium/long run is questionable. The Commission will have to monitor this kind of national scheme very closely to ensure that their positive effects outweigh the distortion of competition in the commercial rental market.

Thus far, only one scheme among those approved under Article 107(3)(b) TFEU in light of the Temporary Framework was intended to support undertakings in coping with a peak in demand for health-related goods. This is the Italian support scheme for the production and supply of medical equipment and masks during the coronavirus outbreak.^91^ Under this scheme, financial support is made available to companies that set up new facilities for or expand the production of medical devices and personal protective equipment (PPE). Another interesting (but isolated) example of a measure aimed at enhancing the resilience of the health sector (beyond the production of health-related goods) is a Dutch scheme providing for direct grants to set up e-health applications.^92^ The Dutch authorities highlight that “the health care sector, providers of social support services, health care services and youth care are confronted with the measures of social distancing and quarantine, which have led to the urgent need to invest in e-health applications^93^ to ensure the provision of these services at home”, and that they “incur extra and unforeseen costs to purchase and implement e-health applications”.^94^ In order to prevent financial difficulties, the scheme provides for grants to be awarded “for activities related to the purchase, lease, licensing and implementation of e-health applications” commenced after 27 February 2020.^95^ The Commission considered the measure necessary to remedy a serious disturbance in the Dutch economy and deemed it appropriate to address the “shortage or unavailability of liquidity *and [preserve] the continuity of economic activity in the health care sector* confronted with the measures of social distancing and quarantine”.^96^

On the whole, the Temporary Framework has facilitated the deployment of State aid to support businesses that are particularly vulnerable to the lockdown measures due to an ensuing lack of demand and the consequent scarcity of liquidity. Some aid schemes, such as those related to rent reliefs, seem to be primarily aimed at stimulating resilience by way of preventing arrears, but their potential for market disruption seems high. Interestingly, Article 107(3)(b) TFEU has not served the purpose of increasing the preparedness of the health sector and has been sparingly used in this context. In that regard, as it will be discussed in the remainder of this article, the role of the derogation provided for in Article 107(3)(c) TFEU has been much more impactful. It is evident from the decisions adopted that the Commission is keen on preventing the collapse of entire segments of the economy put under strain because of the COVID-19 outbreak and on strengthening their resilience in the medium term. While, as yet, the Framework has not been used to allow for the rescue of weak companies, the assessment of the Portuguese aid to TAP under “Rescue and Restructuring Guidelines” and the recent proposal to further extend the scope of the Temporary Framework to enable the provision of aid to small enterprises in difficulty before the pandemic seem to open the door wide to further flexibility, far beyond the rules set out in the current Framework.

## III. The role of EU State aid law in reducing health risks

As noted above, the pandemic and the measures taken to contain the virus have caused a severe shock to the economy. Until the health risks are fully neutralised by making the health sector more resilient and by finding a vaccine and an effective treatment to the deadly disease caused by the virus, the European economy will inevitably continue to stagnate or even contract further. In this context, the Commission has acknowledged that national money could, in tandem with substantial EU funding, make a difference. In that regard, the Commission has recognised that EU State aid rules have great potential to contribute to the management of health risks. For this reason, at the beginning of April, it adopted the first amendment to the Framework and, *inter alia*, increased the opportunities for public support to the research and development (R&D), testing and production of health-related products relevant to combatting COVID-19.^97^ While initially the Temporary Framework had focused exclusively on Article 107(3)(b) TFEU, the Commission instead turned to Article 107(3)(c) TFEU (which allows Member States to provide “aid to facilitate the development of certain economic activities or of certain economic areas, where such aid does not adversely affect trading conditions to an extent contrary to the common interest”)^98^ as a primary means through which it could approve measures aimed at increasing the resilience of the healthcare sector (broadly conceived), as well as enhancing preparedness to respond to the any further threats stemming from the virus.

Under the amended Temporary Framework, the Commission “will consider compatible with the internal market aid for R&D projects carrying out COVID-19 and other antiviral relevant research”,^99^ provided that a number of conditions are met: firstly, the aid must be granted in the form of direct grants, repayable advances or tax advantages by 31 December 2020; secondly, for R&D projects started as of 1 February 2020, the aid is *de jure* deemed to have an incentive effect, while for projects started before that date, the incentive effect is deemed to exist “if the aid is necessary to accelerate or widen the scope of the project”; thirdly, the eligible costs include personnel costs and costs for digital and computing equipment, for diagnostic tools, for data collection and processing tools, for R&D services, for pre-clinical and clinical trials, for obtaining, validating and defending patents and other intangible assets, for obtaining the conformity assessments and/or authorisations necessary for the marketing of new and improved vaccines and medicinal products, medical devices, hospital and medical equipment, disinfectants and PPE; and fourthly, the aid intensity for each beneficiary may cover 100% of eligible costs for fundamental research but cannot exceed 80% of eligible costs for industrial research and experimental development (unless the research project is transnational or is carried out in cross-border collaboration with research organisations or other undertakings). Importantly, the aid approved under this strand may be combined with support from other sources. The aid beneficiary, however, must commit “to grant non-exclusive licences under non-discriminatory market conditions to third parties in the EEA”.^100^

Investment aid, in the form of direct grants, tax advantages or repayable advances “for the construction or upgrade of testing and upscaling infrastructures required to develop, test and upscale, up to first industrial deployment prior to mass production, COVID-19 relevant products” (including vaccines and treatments), will also be considered compatible with the internal market provided that certain conditions laid down in the Framework are met. Notably, among these, it is stipulated that the investment project shall be completed within six months of the date of granting the aid. The aid intensity must also not exceed 75% of the eligible costs (but may be increased by an additional 15 percentage points if the investment is concluded within two months of the date of the aid being granted or the date of the application of the tax advantage, or if the support comes from more than one Member State). However, this aid cannot be combined with other investment aid for the same eligible costs. The services provided by the testing and upscaling infrastructures must be in line with market prices, and those infrastructures shall be open to various users on a non-discriminatory basis.

Beyond the aid currently permitted under the derogation provided in Article 107(3)(c) TFEU, the Temporary Framework has also sought to allow Member States to address any peak in demand among certain health-related goods. In that vein, the Framework clearly acknowledged that the use of Article 107(3)(b) TFEU (as it occurred for the Italian scheme approved in March) was not the most appropriate avenue for allowing this type of measure. The Commission considers investment aid in the form of direct grants, tax advantages or repayable advances for the production of COVID-19-relevant products (such as masks, ventilators, protective clothing and equipment as well as diagnostic tools) compatible with the internal market provided that the aid is granted by 31 December 2020. In line with what was stipulated for aid to R&D projects, the incentive effect is presumed for investment projects started after 1 February 2020. For those projects that commenced prior to this, the aid is deemed to have an incentive effect “if the aid is necessary to accelerate or widen the scope of the project”. The investment project must be completed within six months of the date of granting of the aid. Among the eligible costs, the Commission includes all investment costs necessary for the production of health-related products. In terms of aid intensity, the provision echoes what is provided for in relation to aid for the purposes of R&D: hence, the aid intensity shall not exceed 80% of the eligible costs, but can be increased by a further 15 percentage points if the support comes from more than one Member State, or if “the investment is concluded within two months after the date of the aid granting or the date of application of the tax advantage”. However, it is not possible to combine sources, meaning that aid under this strand may not be added to other investment aid for the same eligible costs.

As yet, a number of countries, after the swift assessment and approval of the Commission, have enacted measures under these amended rules. Several schemes intend to back R&D in conjunction with the production of health-related products.^101^ In some cases, the schemes are forward-looking: this is the case for a Maltese scheme, worth €5.3 million, which is purposely geared towards improving “foresight tools and methodologies for the future, by drawing on lessons learnt from the current pandemic”.^102^ National support for R&D should complement the substantial EU funding awarded to research projects and the funds raised under the so-called “Coronavirus Global Response Initiative” to ensure the collaborative development and universal deployment of diagnostics, treatments and vaccines against coronavirus. Under the latter flagship initiative, the Commission has, at the time of writing, registered €15.9 billion in pledges, which is far beyond the initial target of €7.5 billion.^103^ The use of State aid also tallies with the use of other EU funding in the R&D of new products to fight the virus: for example, the EU has awarded nearly €166 million through the European Innovation Council (EIC) Accelerator Pilot to various companies, which complements the use of Horizon 2020 funding.^104^

On the whole, Article 107(3)(c) TFEU has been identified as the suitable legal basis for dealing with this health crisis. The Commission has tried to channel State aid towards addressing those health risks that are at the heart of the pandemic and whose reduction is key to a return to “normality”. In this respect, it is evident that the Commission has considered State aid control as a useful instrument to complement pan-European solutions.

## IV. Concluding remarks

Long before the pandemic, Biondi and Righini defined State aid as an area “where ‘eternal’ questions about the co-existence of national and supranational elements are particularly pressing, as they impact on core functions of the State and on the regulatory competence of the [EU]”.^105^ They suggest that these conflicting interests have been somewhat “recomposed over time to follow the process of European integration”.^106^ This seems confirmed by the Temporary Framework and, more generally, by the way in which the Commission has used State aid control in the past months. In the current climate, and in light of the rapidly evolving nature of the COVID-19 contagion, EU State aid law has been an important tool in the Commission’s arsenal that is capable of reducing the economic risk associated with the pandemic, with a view to ensuring the survival of the internal market. The Commission has also (at least to some extent) used State aid control to support Member States in mitigating health risks. In substance, the Commission has advocated that Member States could (and should) use public spending to achieve the common European objective of “beating the virus”. In this regard, it transpires that the Commission has used its powers in the area of State aid in conjunction and synergy with other competences, stimulating (if not actively advocating) a more coordinated response at Member State level. This is quite evident, for example, with regard to SMEs. Ultimately, the decision to grant State aid remains a national one, even if the Commission allows it *ex ante*, or even incentivises it. However, it seems that the EU executive has been very determined in using EU State aid law as an important “risk management tool” to tame the COVID-19 crisis, and it has channelled the use of Treaty derogations to address different risks.

By adopting the concept of “risk” as the lens of analysis, this article shows that State aid has proven to be a flexible and yet significant instrument to redress negative consequences deriving from the exposure to lockdowns and to address the vulnerability of certain sectors and increase their resilience, but also to mitigate health risks. This twofold purpose has been highlighted in the recent white paper on foreign subsidies, in which it was explicitly stated that “State aid is an indispensable means at the disposal of public authorities to *stabilise the economy* and *accelerate research in the coronavirus*”.^107^

As of March 2020, various national lockdown measures have paralysed the demand for several goods and services and, consequently, disrupted their supply,^108^ causing an unparalleled economic downturn.^109^ Since then, the Commission has approved more than €1.9 trillion in State aid^110^ that tackles, to varying degrees, what have been identified as the core determinants of the economic risk linked to COVID-19. In particular, this contribution contends that Article 107(2)(b) TFEU has allowed Member States to reduce the “exposure” of certain sectors of the economy to the consequences of the virus itself. In other words, the economic risk determined by a sector or a business being “exposed” has been counteracted through measures that compensate for damage suffered as a result of the lockdown measures adopted by the Member States approved under this *de jure* derogation. Moreover, the Commission, evidently building on the experience gained during the financial crisis of 2008, has passed new horizontal rules in the form of a Temporary Framework, identifying Article 107(3)(b) TFEU as an important legal basis through which States can inject liquidity into national markets in order to protect undertakings and jobs. The analysis conducted demonstrates that the measures considered compatible with the internal market under Article 107(3)(b) TFEU aim to tackle the vulnerability of certain economic sectors or certain undertakings and to increase their overall resilience, improving their potential to weather this period of extreme economic uncertainty. As the discussion conducted in Section III shows, addressing vulnerability and increasing resilience go hand in hand within the Temporary Framework, which is ultimately geared towards ensuring the capacity of certain economic sectors to recover to pre-COVID levels, but also towards stimulating the capacity of market operators to bounce back. The Framework makes it even more evident that, in applying EU State aid rules, the Commission has developed its own vision of “good” aid, *de facto* aligning national State aid policies with the EU’s goals.^111^ For example, the attention paid by the Temporary Framework to SMEs is in line with the overall EU policy in the field.

As predicted,^112^ the Commission has been following a path that is similar (but not identical) to that adopted during the financial crisis of 2008. In that connection, it has resorted to a set of horizontal rules (revised periodically) related to the application Article 107(3)(b) TFEU. While the financial crisis, which occurred in 2008, provoked a tremendous shock to the global economy, its contours were more defined than those of the current pandemic.^113^ By contrast, quite worryingly, it is unclear when (and whether) we could eventually return to our normal lives and at what point undertakings may see a return to “business as usual”, and whether a second wave might prompt further lockdown measures. For this reason, the Commission has resorted to Article 107(3)(c) TFEU to allow Member States to tackle health risks, enable R&D and ensure the supply of products to fight the current outbreak of the coronavirus and a future revamping of the pandemic.

Furthermore, the second amendment to extend the scope of the Temporary Framework has been vital to enabling Member States to put forward public interventions in the form of recapitalisation aid to non-financial companies in need, with the view to helping to reduce the risk to the EU economy as a whole.^114^ While Member States might opportunistically use the Temporary Framework in order to push measures not necessarily prompted by the pandemic, as yet, the Commission seems to have been able to avoid such a use. The Commission proposal to extend the scope of the Framework (currently under discussion) and the recent decision on TAP, however, show a rather flexible approach, which might open the door to relaxed control over schemes to rescue unhealthy companies and will indeed have to be looked at closely in order to avoid unwelcome and unnecessary distortions of competition beyond the scope of the COVID-19 recovery goal.

The approach taken by the Commission has not been without criticism. Hornkohl and van’t Klooster have suggested that “[b]y relaxing State aid rules and clearing measures at an astonishing speed”, the Commission has substantially opened the door to a deepening of economic inequalities among Member States.^115^ They note that the Member States that, thus far, have made massive use of the Temporary Framework are the “wealthy” ones. This criticism echoes Lamadrid and Buendia Sierra’s words.^116^ However, on the one hand, all of the Member States have benefitted, to some degree, from these flexible rules, and, on the other hand, the application of the Framework has contributed to shaping a European answer to pandemic-generated risks. Furthermore, the high probability of asymmetric distortions of competition seems to be addressed (at least partially) by the latest proposal for a pan-European recovery instrument.^117^

On the whole, the Commission has played a fundamental role in allowing Member States to support their economies, while promoting policy coherence across the EU and streamlining the assessment process. In that, the Temporary Framework is consistent with the objectives of the modernisation plan launched in 2012.^118^ While endorsing an increase of public spending so as to achieve an objective of common interest, the Commission has also established itself as a “risk management authority”. It has attempted to work fast, alongside Member States, to ensure that national public support is increasing the resilience of the EU economy as whole and is not endangering the very existence of the internal market during a time in which it is under unprecedented threat. Remarkably, amidst this crisis, the Commission has launched a white paper on foreign subsidies, in which it identifies a regulatory gap in the current State aid legal architecture to address foreign subsidies that distort competition in the internal market. Looking forward to when the pandemic will hopefully be over, it seems likely that new avenues to reinforce and revamp State aid control will be explored. When aid is progressively unwound, the Commission will have to ensure further coordination in order to avoid additional asymmetric distortion to competition in the EU, which makes a discussion on how State aid measures will have to be phased out a key priority for 2021.

## Footnotes

1 HW Micklitz, “The Corona Threat – An Opportunity to Rethink the European Economic Constitution and European Private Law” (2020) 11 European Journal of Risk Regulation 249.

2 For a discussion on possible economic scenarios, see N Fernandes, “Economic Effects of Coronavirus Outbreak (COVID-19) on the World Economy” (22 March 2020) <https://ssrn.com/abstract=3557504>. For a more general overview of the economic impact of the pandemic, see A Delivorias and N Scholz, “Economic impact of epidemics and pandemics”, EPRS – European Parliamentary Research Service (February 2020) <https://www.europarl.europa.eu/RegData/etudes/BRIE/2020/646195/EPRS_BRI(2020)646195_EN.pdf>.

3 See <https://ec.europa.eu/info/live-work-travel-eu/health/coronavirus-response_en>. For a commentary, see KP Purnhagen et al, “More Competences than You Knew? The Web of Health Competences for Union Action in Response to the COVID-19 Outbreak” (2020) 11 European Journal of Risk Regulation 297.

4 L Garicano, “The COVID-19 bazooka for jobs in Europe”, in R Baldwin and B Weder di Mauro, *Mitigating the COVID Economic Crisis: Act Fast and Do Whatever It Takes*, A VoxEU.org Book (London, CEPR Press 2020) 129, at 130.

5 R Baldwin and B Weder di Mauro, “Introduction” in R Baldwin and B Weder di Mauro, *Mitigating the COVID Economic Crisis: Act Fast and Do Whatever It Takes*, A VoxEU.org Book (London, CEPR Press 2020) 1, at 19 et seq.

6 A Biondi, “State Aid in the Time of COVID-19” (*EU Law Live*, 25 March 2020) <https://eulawlive.com/op-ed-state-aid-in-the-time-of-covid-19-by-andrea-biondi/>. Rosanò contends that the Commission has approved State aid schemes in forty-eight hours and argues that this speediness is possible because of the “significant expertise” accumulated after the economic crisis of 2008 (A Rosanò, “Adapting to Change: COVID-19 as a Factor Shaping EU State Aid Law”, European Papers – European Forum, 7 May 2020).

7 See, among many others, SA.57552 (2020/N) Compensation scheme agricultural and horticultural undertakings COVID-19 – NL – Amendment SA.57217, C(2020) 4017 final, in which “The Netherlands exceptionally agree to waive their rights deriving from Article 342 of the Treaty on the Functioning of the European Union (‘TFEU’), in conjunction with Article 3 of Regulation 1/19581 and to have this Decision adopted and notified in English”.

8 On the concept of State aid, see *inter alia* JJ Piernas Lopez, “The Evolving Nature of the Notion of Aid under EU Law” (2016) 3 European State Aid Law Quarterly 400; and A Biondi and O Stefan, “The Notice on the Notion of State Aid: Every Light has its Shadow” in B Nascimbene and A. Di Pascale, *The Modernisation of EU State Aid Control – Evolution and Perspectives of the EU Rules on State Aids and Services of General Economic Interest* (Berlin, Springer 2018) pp 43–61.

9 Communication from the Commission to the European Parliament, the European Council, the Council, the European Central Bank, the European Investment Bank and the Eurogroup, “Coordinated economic response to the COVID-19 Outbreak”, COM(2020)112 final.

10 On the State aid regime, see generally HCH Hofmann and C Micheau (eds), *State Aid Law of the European Union* (Oxford, Oxford University Press 2016).

11 Commission Regulation (EU) 651/2014 of 17 June 2014 declaring certain categories of aid compatible with the internal market in application of Articles 107 and 108 of the Treaty [2014] OJ L 187/1.

12 Communication from the Commission, “Temporary Framework for State aid measures to support the economy in the current COVID-19 outbreak 2020/C 91 I/01 C/2020/1863”, [2020] OJ C 91I/1 (Temporary Framework). A consolidated version of the Temporary Framework is available at <https://ec.europa.eu/competition/state_aid/what_is_new/TF_consolidated_version_as_amended_3_april_and_8_may_2020_en.pdf>. On the Temporary Framework, see Rosanò, supra, note 6. Among the various commentaries and summaries, see M Nissen and J Rivas, “The EU Commission adopts a new Temporary Framework” <https://www.concurrences.com/en/bulletin/news-issues/preview/the-eu-commission-adopts-a-new-temporary-framework-to-complement-the-existing-en>; P Riedel, T Wilson and S Cranley, “EU State Aid and COVID-19” (*Kluwer Competition Law Blog*, 24 March 2020) <http://competitionlawblog.kluwercompetitionlaw.com/2020/03/24/eu-state-aid-and-covid-19/>; M Motta and M Peitz, “EU state aid policies in the time of COVID-19” (*VOX CEPR Policy Portal*, 18 April 2020) <https://voxeu.org/article/eu-state-aid-policies-time-covid-19>.

13 Para 10 of the Temporary Framework (consolidated version).

14 Paras 17–18 of the Temporary Framework (consolidated version).

15 JL Buendia Sierra and A Dovalo, “State Aid Versus COVID-19: The Commission Adopts a Temporary Framework” (2020) 1 European State aid Law Quarterly 3.

16 Para 91 of the Temporary Framework (consolidated version).

17 Communication from the Commission, “Amendment to the Temporary Framework for State aid measures to support the economy in the current COVID-19 outbreak 2020/C 112 I/01 C/2020/2215”, [2020] OJ C 112I/1.

18 Communication from the Commission, “Amendment to the Temporary Framework for State aid measures to support the economy in the current COVID-19 outbreak”, C(2020) 3156 final <https://ec.europa.eu/commission/presscorner/detail/en/ip_20_838>.

19 See the Commission Statement on consulting Member States on the proposal to expand the State aid Temporary Framework to further support micro, small and start-up companies and incentivise private investments, STATEMENT/20/1054 <https://ec.europa.eu/commission/presscorner/detail/en/STATEMENT_20_1054>.

20
ibid.

21 I Noy et al, “The economic risk from COVID-19 is not where COVID-19 is” (*VOX CEPR Policy Portal*, 1 May 2020) <https://voxeu.org/article/economic-risk-covid-19-not-where-covid-19>.

22
ibid.

23
ibid.

24 The expression was used by J Almunia, Speech “Banking crisis, financial stability and State aid: The experience so far” <https://ec.europa.eu/commission/presscorner/detail/en/SPEECH_13_223>. The potential role of the Commission was already discussed by JJ Piernas Lopez, “State aid and COVID-19 outbreak – What can we expect?” (*EU Law Live*, 17 March 2020) <https://eulawlive.com/op-ed-state-aid-and-covid-19-outbreak-what-can-we-expect-by-juan-jorge-piernas-lopez/>.

25 See <https://medium.com/sequoia-capital/coronavirus-the-black-swan-of-2020-7c72bdeb9753>. For a comment on this aspect, see A Renda and R Castro, “Towards Stronger EU Governance of Health Threats after the COVID-19 Pandemic” (2020) 11 European Journal of Risk Regulation 273.

26 W Devroe and N Colpaert, “Corona and EU economic law: Free movement of goods” <https://coreblog.lexxion.eu/corona-and-eu-economic-law-free-movement-of-goods/>.

27 Nicolaides reports that the number of approved and published COVID-19 measures, as of 10 July 2020, amounts to 202. See in that regard <https://www.lexxion.eu/en/stateaidpost/2019-competition-report/#_ftn1>.

28 Emphasis added.

29 Guidelines for State aid in the agricultural and forestry sectors and in rural areas 2014 to 2020 [2014] OJ C 204/1, para 34 and paras 327 et seq.

30 The Court of Justice of the EU (CJEU) has consistently held that derogations must be interpreted narrowly, and, in line with this general principle, the concepts of “natural disaster” and “exceptional occurrence” must be interpreted strictly. See *inter alia* Case C-73/03 *Spain v. Commission*, ECLI:EU:C:2004:711, para 37.

31 Guidelines for State aid in the agricultural and forestry sectors and in rural areas 2014 to 2020 [2014] OJ C 204/1, para 330.

32 Joint decision on State aid schemes N 299/1996, N 290/1996, N 178/1996 and N 289/1996 (approved by Commission letter SG(1996) D/05081 of 15 March 1996) and decision on State aid scheme N 795/1996 (approved by Commission letter SG(1998) D/01616 of 21 January 1998).

33 For a discussion of aid considered compatible under Article 107(2) TFEU, see P Nicolaides, “Application of Article 107(2)(b) TFEU to Covid-19 Measures: State Aid to Make Good the Damage Caused by an Exceptional Occurrence”, (2020) Journal of European Competition Law & Practice lpaa026.

34
*Inter alia* Joined Cases C-346/03 and C-529/03, *Giuseppe Atzeni* ECLI:EU:C:2006:130, para 79.

35 SA.57051 – COVID 19 – Aid for cancelled or postponed cultural events in Sweden, C(2020) 2643 final, paras 51 et seq.

36
ibid.

37 SA.56685 (2020/N) – DK – Compensation scheme for cancellation of events related to COVID-19, C(2020) 1698 final.

38
ibid. See also the most recent SA.57930 Temporary targeted compensation scheme for companies affected by COVID 19 prohibitions (bans and cancelled events) approved on 13 July 2020.

39 SA.57051 COVID 19 – Aid for cancelled or postponed cultural events in Sweden C(2020) 2643 final.

40 SA.56774 (2020/N) – DK – Compensation for companies particularly exposed to COVID-19 outbreak, C(2020) 2294 final, para 4. Emphasis added. This latter scheme was first approved on 8 April 2020, then prolonged and further widened in early May (SA.57151 (2020/N) – Denmark – Amendment and prolongation of compensation scheme SA.56774 for companies particularly exposed to COVID-19 outbreak, C(2020) 3041 final). See also SA.57284 (2020/N) – Finland COVID-19: Finnish damage compensation scheme for restaurants C(2020) 3607 final. Another example is the Danish “Temporary wage compensation scheme for self-employed financially affected by the COVID-19” (SA.56791 (2020/N) – Denmark – Temporary wage compensation scheme for self-employed financially affected by the COVID-19 C(2020) 1982 final). The scheme was extended in May. See SA.57199 (2020/N) – Denmark – Amendment to State Aid SA.56791 (2020/N) on temporary wage-compensation scheme for self-employed financially affected by the COVID-19, as amended by State aid SA.56892 (2020/N), C(2020) 3099 final.

41 SA.57217 (2020/N) NL LNV AGRI Compensation scheme agricultural and horticultural undertakings COVID-19, C(2020) 3143 final. This scheme was amended in June: SA.57552 (2020/N) Compensation scheme agricultural and horticultural undertakings COVID-19 – NL - Amendment SA.57217, C(2020) 4017 final.

42 SA.57291 (2020/N) – Austria – COVID-19; Compensation Scheme: Directive for fixed cost subsidies C(2020) 3483 final.

43 Those measures were not unpredicted. By contrast, various journals reported that, at the beginning of March, Olivier Guersent, director-general for competition, had already predicted it “quite likely that all of the airlines will suffer a lot from this crisis” and that they “will need to be compensated simply so that they don’t find themselves in a very difficult situation solely because of the crisis” (T Powely and J Espinosa, “Airlines step up calls for emergency state aid” (*Financial Times*, 15 March 2020) <https://www.ft.com/content/97c14000-66dc-11ea-800d-da70cff6e4d3>).

44 SA.56867 COVID 19 – Support for Condor. See press release <https://ec.europa.eu/commission/presscorner/detail/en/IP_20_752>.

45 SA.56795 Compensation for the damage caused by the COVID-19 outbreak to Scandinavian Airlines. See press release <https://ec.europa.eu/commission/presscorner/detail/en/IP_20_667>.

46 SA.56765 COVID-19 *Moratoire sur le paiement de taxes et redevances aéronautiques en faveur des entreprises de transport public aérien sous licences d”exploitation délivrées par la France*, C(2020) 2097 final.

47 See <https://ec.europa.eu/commission/presscorner/detail/en/IP_20_1275>.

48 Communication from the Commission – Guidelines on State aid for rescuing and restructuring non-financial undertakings in difficulty [2014] OJ C 249/1. The Rescue and Restructuring Guidelines have enabled Member States to put in place support schemes for SMEs (on this point, see also Annex 3 to the Communication from the Commission to the European Parliament, the European Council, the Council, the European Central Bank, the European Investment Bank and the Eurogroup, “Coordinated economic response to the COVID-19 Outbreak”, COM(2020)112 final).

49 Nicolaides, supra, note 33.

50 In fact, few measures were adopted under Article 107(3)(b) TFEU, such as a Belgian guarantee scheme to support companies affected by the coronavirus outbreak (State Aid SA.56819 – Belgium – COVID-19 – Loan guarantee scheme in response to the COVID-19 crisis, C(2020) 2364 final).

51 Communication from the Commission to the European Parliament, the European Council, the Council, the European Central Bank, the European Investment Bank and the Eurogroup, “Coordinated economic response to the COVID-19 Outbreak”, COM(2020)112 final.

52 Para 9 of the Temporary Framework (consolidated version). Emphasis added.

53 Paras 24 et seq. of the Temporary Framework (consolidated version).

54 Paras 26 et seq. of the Temporary Framework (consolidated version).

55 Para 28 of the Temporary Framework (consolidated version).

56 Para 32 of the Temporary Framework (consolidated version).

57 Paras 40 et seq. of the Temporary Framework (consolidated version). This part was added in April in the first set of amendments to the Temporary Framework (see <https://ec.europa.eu/commission/presscorner/detail/en/ip_20_570>).

58
ibid.

59 Para 43 of the Temporary Framework (consolidated version).

60 For a list, see the summary “Coronavirus Outbreak – List of Member State Measures approved under Article 107(2)(b) TFEU, Article 107(3)(b) TFEU and under the Temporary State Aid Framework” <https://ec.europa.eu/competition/state_aid/what_is_new/State_aid_decisions_TF_and_107_2_b_and_107_3_b.pdf>.

61 A definition of SMEs is included in Annex I of the GBER, to which most national schemes refer.

62 SA.56690 (2020/N) – Italy State guarantee to support debt moratorium by banks to SME borrowers under the Temporary Framework for State aid measures to support the economy in the current COVID-19 outbreak C(2020) 1984 final.

63 SA.56708 (2020/N) – Denmark Danish guarantee scheme for SMEs affected by COVID-19, C(2020) 1885 final.

64 See, eg, SA.56996 (2020/N) – Poland COVID-19: repayable advance scheme for micro, small and medium sized enterprise C(2020) 2822 final. SA.56839 (2020/N) – Greece COVID-19: Support to SME loan obligations in the form of grants under the Temporary Framework for State aid measures to support the economy in the current COVID-19 outbreak, C(2020) 2348 final.

65 SA.57132 (2020/N) – Belgium COVID-19: Flemish subordinated loan scheme for start-ups, scale-ups and SMEs C(2020) 3039 final. See also: SA.57246 (2020/N) – Belgium COVID-19: Second Flemish subordinated loan scheme for start-ups, scale-ups and SMEs, C(2020) 3198 final. Other measures include: SA.57066 (2020/N) – Lithuania COVID-19: Direct grants to cover interest on loans of SMEs active in road freight transport, C(2020) 2775 final.

66 See <https://ec.europa.eu/commission/presscorner/detail/en/IP_20_848>.

67 SA.57283 (2020/N) – Bulgaria State aid in the form of direct grants under Operational Programme Innovation and Competitiveness 2014-2020 to support micro and small enterprises to overcome the economic impact of the COVID-19 pandemic outbreak C(2020) 3259 final. The support measures available under the scheme will be to a large part co-financed by the European Regional Development Fund (€75 million).

68 SA.57509 (2020/N) – Ireland COVID-19: Restart Grant, C(2020) 3733 final.

69 SA.57397 (2020/N) – The Netherlands COVID-19: Dutch temporary guarantee scheme for small bank loans for medium sized and small enterprises due to the COVID-19 outbreak, C(2020) 3573 final.

70 State Aid SA.57712 (2020/N) – The Netherlands COVID-19: direct grant scheme to support the fixed costs for small and medium-sized enterprises affected by the COVID-19 outbreak, C(2020) 4442 final.

71 See, eg, SA.57304 (2020/N) and SA.57338 (2020/N) – Luxembourg COVID-19: Solidarity Fund for undertakings affected by the COVID19 outbreak and Aid for commercial shops affected by the COVID-19 outbreak, C(2020) 3629 final.

72 Regulation (EU) No 1287/2013, establishing a Programme for the Competitiveness of Enterprises and small and medium-sized enterprises (COSME) [2013] OJ L347/33.

73 Technopolis, “Interim Evaluation of the COSME Programme – Final Report” (Directorate-General for Internal Market, Industry, Entrepreneurship and SMEs – European Commission, 2017).

74 Committee of the Regions, “EU policy framework on SMEs: state of play and challenges” (2019) <https://cor.europa.eu/en/engage/studies/Documents/EU-SMEs/EU-policy-SMEs.pdf>.

75
ibid.

76 Commission, “A Strong Social Europe for Just Transitions”, COM(2020) 14 final.

77 See supra, note 19.

78
ibid.

79 Commission, “An SME Strategy for a sustainable and digital Europe”, COM(2020) 103 final.

80 See supra, note 64. See, in particular, para 16 of the Commission decision.

81 The EU list of non-cooperative jurisdictions was revised in February by the Economic and Financial Affairs Council (Doc. 6050/20 FISC 61 ECOFIN 82, Brussels, 18 February 2020 <https://data.consilium.europa.eu/doc/document/ST-6050-2020-ADD-2-COR-1/en/pdf>).

82 See supra, note 65. See, in particular, para 55 of the Commission decision.

83 On this issue, see generally I Richelle, W Schön and E Traversa, *State Aid Law and Business Taxation* (Berlin, Springer 2016). Safeguarding a level playing field for companies when it comes to taxation is a priority for the Commission, and this has also been made clear in the Report on Competition Policy 2019, which was released at the beginning of July (Commission, “Report on Competition Policy 2019”, COM(2020) 302 final).

84 Communication from the Commission – Guidelines on State aid for rescuing and restructuring non-financial undertakings in difficulty [2014] OJ C 249/1. The Rescue and Restructuring Guidelines have enabled Member States to put in place support schemes for SMEs (on this point, see also Annex 3 to the Communication from the Commission to the European Parliament, the European Council, the Council, the European Central Bank, the European Investment Bank and the Eurogroup, “Coordinated economic response to the COVID-19 Outbreak”, COM(2020)112 final).

85 See <https://ec.europa.eu/commission/presscorner/detail/en/ip_20_1029>.

86 See <https://ec.europa.eu/commission/presscorner/detail/en/IP_20_349>.

87 See supra, note 19.

88 SA.57135 (2020/N) – Lithuania COVID-19: Partial Rent Compensation for the Enterprises Most Affected by COVID-19 C(2020) 2957 final.

89 SA.57143 (2020/N) – Slovenia COVID-19: Liquidity guarantee scheme and rent relief, C(2020) 2968 final.

90 A summary of those decisions can be found at <https://ec.europa.eu/info/live-work-travel-eu/health/coronavirus-response/jobs-and-economy-during-coronavirus-pandemic/state-aid-cases_en>. See also SA.57599 (2020/N) – Slovak Republic COVID-19: Rent rebates for tenants.

91 See <https://ec.europa.eu/commission/presscorner/detail/en/IP_20_507>.

92 SA.56915 (2020/N) – The Netherlands Direct grant scheme for e-Health services at home under the Temporary Framework for State aid measures to support the economy in the current COVID-19 outbreak, C(2020) 2228 final.

93
ibid. These e-health applications are “apps to ensure proper drug dosage and usage (managing medication) or apps for wound care management to enable digital wound care. E-health applications enable patients to stay at home longer. They contribute to the continuity of support or remote care for patients that have to stay at home”.

94
ibid, para 4.

95
ibid, para 17.

96
ibid, para 34. Emphasis added.

97 Support cannot be granted to undertakings that were experiencing difficulties (such as accumulated losses) or were subject to insolvency procedures at the end of 2019. The Framework refers to the notion of undertaking in difficulty provided for in the GBER (see paras 46 et seq. of the Temporary Framework – consolidated version).

98 Existing guidelines (see supra, note 48) already support the assessment of national measures notified under the remit of this provision that are aimed at helping companies in need of urgent rescue aid.

99 Paras 35 et seq. of the Temporary Framework (consolidated version).

100
ibid.

101 SA.56954 (2020/N) – Luxembourg – Scheme for COVID-19 related R&D aid and investment aid for the production of COVID-19 relevant products, C(2020) 2351 final. See also State aid SA.57100 (2020/N) – Germany – COVID-19 – Federal Framework Scheme “Aid for Covid-19 related R&D, investments in testing infrastructures and production facilities” (“*Bundesregelung Forschungs-, Entwicklungs- und Investitionsbeihilfen*”) C(2020) 2871 final; SA.56961 (2020/N) – Czech Republic – Investment aid scheme for COVID-19 relevant products; SA.57453 (2020/N) – Ireland COVID-19 – Scheme to facilitate COVID-19 relevant research and development, to support construction and upgrade of testing and upscaling facilities for COVID-19 relevant products and to support investments into the production of COVID-19 relevant products C(2020) 3726 final; SA.57367 (2020/N) – France COVID-19: Aid for COVID-19 relevant R&D projects, investment into relevant testing and upscaling infrastructures, and investment into COVID-19 relevant production capacities.

102 See <https://ec.europa.eu/commission/presscorner/detail/en/IP_20_725>.

103 See <https://global-response.europa.eu/index_en>.

104 See <https://ec.europa.eu/commission/presscorner/detail/en/ip_20_1007>

105 A Biondi and E Righini, “An Evolutionary Theory of EU State Aid Control”, in D Chalmers and A Arnull, *The Oxford Handbook of European Union Law* (Oxford, Oxford University Press 2015) p 688.

106
ibid, p 689.

107 Commission, “White Paper on levelling the playing field as regards foreign subsidies” COM(2020) 253 final. Emphasis added.

108 See <https://ec.europa.eu/commission/presscorner/detail/en/statement_20_587>.

109 Communication from the Commission Temporary Framework for State aid measures to support the economy in the current COVID-19 outbreak [2020] OJ C 91I/1, para 2.

110 See <https://ec.europa.eu/commission/presscorner/detail/en/IP_20_838>.

111 D Ferri and JJ Piernas Lopez, “The Social Dimension of EU State Aid Law and Policy” (2019) 21 Cambridge Yearbook of European Legal Studies 1.

112 JJ Piernas Lopez, “State aid and COVID-19 outbreak – What can we expect?” (*EU Law Live*, 17 March 2020) <https://eulawlive.com/op-ed-state-aid-and-covid-19-outbreak-what-can-we-expect-by-juan-jorge-piernas-lopez/>.

113 Micklitz, supra, note 1.

114 Amendment to the Temporary Framework for State aid measures to support the economy in the current COVID-19 outbreak C(2020) 3156 final.

115 L Hornkohl and J van’t Klooster, “With Exclusive Competence Comes Great Responsibility” (*Verfassungblog*, 29 April 2020) <https://verfassungsblog.de/with-exclusive-competence-comes-great-responsibility/>.

116 In this respect, see A Lamadrid and JL Buendía Sierra, “A Moment of Truth for the EU: A Proposal for a State Aid Solidarity Fund” (2020) 11 Journal of European Competition Law & Practice 1.

117 See <https://ec.europa.eu/commission/presscorner/detail/en/ip_20_940>.

118 Commission, “EU State Aid Modernisation (SAM)”, COM(2012) 209 final.

